# Exploration of Symptom Scale as an Outcome for Deprescribing: A Medication Review Study in Nursing Homes

**DOI:** 10.3390/ph15050505

**Published:** 2022-04-21

**Authors:** Dagmar Abelone Dalin, Sara Frandsen, Gitte Krogh Madsen, Charlotte Vermehren

**Affiliations:** 1 Department of Clinical Pharmacology, Copenhagen University Hospital Bispebjerg, DK-2400 Copenhagen, Denmark; dagmar.abelone.dalin@regionh.dk (D.A.D.); sara.frandsen@regionh.dk (S.F.); 2 General practice ”Roskilde Lægehus”, Roskilde, DK-4000 Roskilde, Denmark; gitte.madsen@dadlnet.dk; 3 Department of Drug Design and Pharmacology, PHARMA, Faculty of Health and Medical Sciences, University of Copenhagen, DK-2100 Copenhagen, Denmark

**Keywords:** medication review, deprescriptions, quality of life, aged, aged, 80 and over, nursing homes, deprescribing

## Abstract

The use of inappropriate medication is an increasing problem among the elderly, leading to hospitalizations, mortality, adverse effects, and lower quality of life (QoL). Deprescribing interventions (e.g., medication reviews (MRs)) have been examined as a possible remedy for this problem. In order to be able to evaluate the potential benefits and harms of a deprescribing intervention, quality of life (QoL) has increasingly been used as an outcome. The sensitivity of QoL measurements may, however, not be sufficient to detect a change in specific disease symptoms, e.g., a flair-up in symptoms or relief of side effects after deprescribing. Using symptom assessments as an outcome, we might be able to identify and evaluate the adverse effects of overmedication and deprescribing alike. The objective of this study was to explore whether symptom assessment is a feasible and valuable method of evaluating outcomes of MRs among the elderly in nursing homes. To the best of our knowledge, this has not been investigated before. We performed a feasibility study based on an experimental design and conducted MRs for elderly patients in nursing homes. Their symptoms were registered at baseline and at a follow-up 3 months after performing the MR. In total, 86 patients, corresponding to 68% of the included patients, received the MR and completed the symptom questionnaires as well as the QoL measurements at baseline and follow-up, respectively. Forty-eight of these patients had at least one deprescribing recommendation implemented. Overall, a tendency towards the improvement of most symptoms was seen after deprescribing, which correlated with the tendencies observed for the QoL measurements. Remarkably, deprescribing did not cause a deterioration of symptoms or QoL, which might otherwise be expected for patients of this age group, of whom the health is often rapidly declining. In conclusion, it was found that symptom assessments were feasible among nursing home residents and resulted in additional relevant information about the potential benefits and harms of deprescribing. It is thus recommended to further explore the use of symptom assessment as an outcome of deprescribing interventions, e.g., in a controlled trial.

## 1. Introduction

The prevalence of chronic diseases increases with age, and the elderly therefore have a higher risk of polypharmacy [1,2]. Polypharmacy is often defined as taking at least five different medications regularly [2,3] and has been shown to increase the risk of inappropriate medication and hospitalizations among elderly patients [4,5,6]. In addition, the risk of adverse effects and drug interactions [2,5,7], mortality [8], and reduced quality of life (QoL) [9] have been correlated with polypharmacy.

Medications that were appropriate at the time of prescription may become inappropriate over time and with age, either because the patient’s health condition improves or the harms outweighs the benefits [10,11]. As an example of the latter, the use of preventive medication, e.g., statins, in the last years of life can cause considerable muscle pain without any preventive effect being expected [12].

Medication review (MR) interventions are considered to be a valuable tool to combat inappropriate polypharmacy through deprescribing recommendations [13]. Deprescribing is defined as the planned and supervised process of dose reduction or stopping of medications that might be causing harm, or which may no longer have a benefit [14]. During an MR, the patient’s complete medication list is critically and structurally reviewed in relation to indications, effects, side effects, and compliance. In MR studies, outcomes are frequently either medication-related, e.g., a decrease in the number of medications, or resource-related, e.g., cost, number of general practitioner (GP) visits, or hospitalization, rather than patient-related [15,16,17]. The use of patient-related outcomes such as QoL is steadily increasing in medication review studies [15] to investigate whether the intervention brings a relevant benefit to the patient. Currently, there is no convincing evidence that a medication review leads to an improved QoL [18]. The QoL scales might not have sufficient sensitivity to be able to detect improvements in QoL, especially not among nursing home residents, for whom a natural rapid deterioration in their condition is expected. However, it is crucial to be able to assess whether a medication review leads to improved patient outcomes upon deprescribing medications. A recent review analyzed the various outcomes of MR studies and found a lack of reporting of potential harm (e.g., adverse events) caused by the interventions [15], making it difficult to evaluate the benefit–risk ratio of MRs. It is our hypothesis that the assessment of symptoms as an outcome of MR could be a feasible and valuable additional approach in order to identify and report the potential benefits and harms of MR. Some specific symptoms, e.g., dizziness, have been used as outcomes in MR, but to our knowledge a systematic symptom assessment has not previously been used as an outcome in MR. The objective of this study was to explore whether symptom assessment is a feasible and a potential valid outcome measure of deprescribing when performing MRs of elderly patients in nursing homes. Additionally, symptom measurements are compared with QoL values in order to investigate whether symptom measurements can contribute further knowledge about the patient-relevant effects of MRs.

## 2. Results

### 2.1. Patients

**Figure 1:**
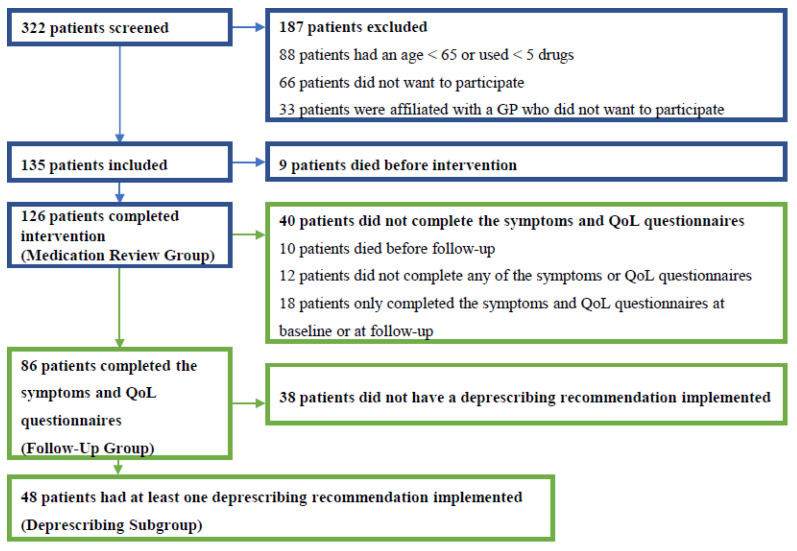
The flow of patients from screening to the inclusion of eligible patients in the Medication Review, Follow-Up, and Deprescribing Subgroup, respectively. The upper five boxes (blue) describe the inclusion process, as well as reasons for exclusion and dropout, whereas the lower four boxes (green) describe the subsets of patients relevant to the aim of this article, i.e., patients who completed the symptoms and quality of life (QoL) questionnaires both at baseline and follow-up were included in the Follow-Up Group. Subsequently, patients in the Follow-Up Group who had a deprescribing recommendation implemented were included in the Deprescribing Subgroup.

In Hvidovre Municipality, Denmark, 322 residents living in the three participating nursing homes were screened for participation in the project. Of these residents, 234 were found to be eligible according to the inclusion criteria. Of these, 135 patients were included in the study (see for further information on inclusion and exclusion). Nine patients (6.7%) died before the intervention; hence, 126 patients in total received the MR intervention. Ten patients (7.9%) died before follow-up and 30 patients (23.8%) did not complete the symptom and QoL questionnaires, resulting in 86 patients who completed the questionnaires both at baseline and follow-up, and 48 patients who had a deprescribing intervention implemented and who completed the symptom and QoL questionnaires (Figure 1).

The participating patients were divided into three investigational groups according to the degree of their study participation: the Medication Review Group (patients who received an MR), the Follow-Up Group (patients who, in addition, completed the symptom and QoL questionnaires), and the Deprescribing Subgroup (patients who had a deprescribing recommendation implemented and who completed the symptom and QoL questionnaires), respectively.

The 126 patients included in the Medication Review Group had an average age of 82 years (SD 7.8) and 32% were male. They had a total of 1575 medications and were affiliated with 18 different general practices. On average, each patient had a mean of 13 medications at baseline, of which 10 were regular medications, and 3 were as-needed medications. Table 1 summarizes the patient characteristics regarding age, medication, and scoring of symptoms and QoL, respectively, divided into the three investigational groups.

### 2.2. Medication Review

We recommended changes to 491 medications (31% of all medications) out of the total of 1.575 medications. The GPs agreed upon 460 of the recommendations (94% of recommendations), and 196 recommendations were implemented at follow-up (45%), of which 159 were deprescribing recommendations. For the 86 patients with completed QoL and symptoms’ questionnaires (the Follow-Up Group), 55 patients had at least one implemented recommendation (both deprescribing and other recommendations) at follow-up, and 48 patients had at least one deprescribing recommendation implemented (the Deprescribing Subgroup). Deprescribing was the most frequent recommendation (78% of all recommendations), and we recommended deprescribing 24% of all 1575 medications. The GPs agreed to deprescribe 23% of all 1575 medications.

### 2.3. Symptoms

For the Follow-Up Group (*n* = 86), there was no significant difference in the mean of symptom scores before and after the intervention: pain (−0.34), tiredness (0.10), drowsiness (0.03), nausea (0.14), loss of appetite (0.42), shortness of breath (−0.03), depression (0.30), and anxiety (−0.02).

**Figure 2:**
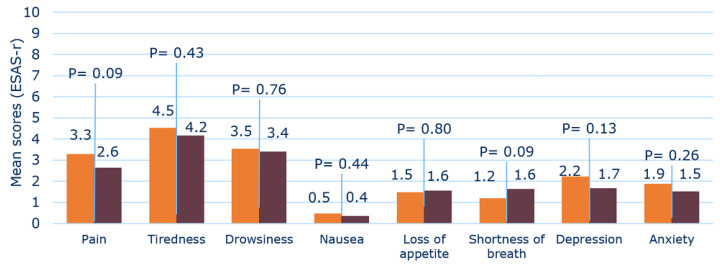
Mean scores of the Deprescribing Subgroup (*n* = 48) for each symptom, measured using the Edmonton Symptom Assessment System (revised version) (ESAS-r) scale, before deprescribing (orange) and after deprescribing (brown). The ESAS-r scale uses a numeric scale from 0–10, where 0 indicates no symptoms and 10 indicates the worst intensity of a symptom. There was no significant difference for any symptoms.

For the Deprescribing Subgroup (*n* = 48), no significant difference in the mean of symptom scores was found either. However, in this group a tendency towards an improvement of the average score within all symptoms, except loss of appetite and shortness of breath, was observed. The average symptom scores for the Deprescribing Subgroup before and after MR, i.e., deprescribing, are shown in Figure 2.

**Figure 3:**
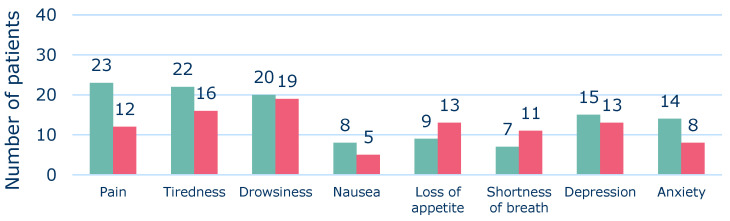
Number of Deprescribing Subgroup patients (*n* = 48) with improvements (green) and deteriorations (red), respectively, in each symptom after deprescribing.

The results suggested that, among the patients in the Deprescribing Subgroup, there was a greater tendency towards the improvement of symptoms compared to a deterioration (Figure 3). Loss of appetite and shortness of breath were the only symptoms among patients of this group for which fewer patients achieved an improvement than a deterioration (Figure 3).

**Figure 4:**
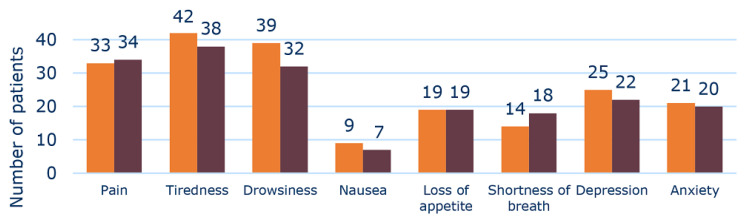
Number of Deprescribing Subgroup patients (*n* = 48) with any presentation of each symptom (answers higher than 0 on the Edmonton Symptom Assessment System (revised version) scale) before (orange) and after (brown) deprescribing. There was no significant difference for any symptoms.

The figure below shows the number of patients who exhibited the individual symptoms (Figure 4) to some degree before and after deprescribing, respectively. The majority of patients suffered from tiredness, drowsiness, and pain. These data show a general tendency that there were no more patients who developed symptoms after deprescribing, apart from symptoms regarding shortness of breath.

One of the purposes of this work was to consider whether symptom assessment could contribute to additional knowledge about the patient-relevant effects of MRs. Hence, we tried to elucidate whether deprescribing of a medication related to a symptom, e.g., pain, had an influence on the symptom score for pain. Overall, we found no tendency for a deterioration of symptoms caused by deprescribing a drug. However, we found a tendency towards improvement for some symptoms. As an example, deprescribing of pain relievers (NSAIDs, paracetamol, opioids, or muscle relaxants) was performed for 12 patients. Of these, seven patients obtained a better pain score, four patients revealed unchanged pain scores, and only one patient experienced an increase in pain at follow-up.

### 2.4. Quality of Life

Overall, only a small change in the EQ-5D index and VAS score was observed during the study period, as shown in Table 2.

For both the Follow-Up Group and the Deprescribing Subgroup, small insignificant changes in the EQ-5D index were found. The overall Follow-Up Group revealed a small insignificant deterioration, whereas the Deprescribing Subgroup revealed a small insignificant improvement. Both groups showed a small insignificant improvement in the VAS score. The improvement was largest in the Deprescribing Subgroup, though it was smaller than the minimal clinically relevant difference of eight [19].

## 3. Discussion

We explored the feasibility of using symptom assessment as an outcome of MRs among nursing home residents with polypharmacy using the ESAS-r [20] in comparison with a QoL assessment, i.e., EQ-5D [21]. One hundred and twenty-six patients met our inclusion criteria, were included in the study, and received MR (the Medication Review Group). However, only 86 patients completed the ESAS-r and EQ-5D (the Follow-Up Group). The primary barriers in the workflow, which prevented a full-sized Follow-Up Group, were related partly to the physical and mental health of the patients and to lack of resources among the nursing staff to support the patients in answering the questionnaires. The allocation of additional support to healthcare providers in nursing homes would probably improve the follow-up rate significantly.

The patient characteristics (Table 1) revealed a patient group which was comparable to other polypharmacy patient groups in nursing homes, although the prevalence of polypharmacy among the residents in this study was higher [22]. It was observed that the patients from the Deprescribing Subgroup on average used two drugs more than patients in the overall Follow-Up Group, i.e., 14 vs. 12 medications per patient. Similarly, the patients from the Deprescribing Subgroup were recommended one more medication change on average compared to the patients in the overall Follow-Up Group, indicating a higher degree of overmedication among the subgroup that had at least one drug deprescribed.

During the MR, deprescribing was by far the most frequent recommendation. The GPs thus agreed that 23% of all medications should be deprescribed. This was consistent with other MR studies for elderly polypharmacy patients, in which deprescribing made up around 15–32% of all recommended changes [23,24,25,26,27,28]. In a previous study, we found that GPs agreed to deprescribe 18% of all medications [29]. Likewise, a Dutch study showed that 13% of all medications had been deprescribed 1 week after an MR [30].

In the present study, the GPs accepted the majority (94%) of the recommendations suggested by the medication team. Of all the accepted recommendations, 45% were implemented at follow-up. This was the case for 35% of the deprescribing recommendations. Thus, the implementation rate was low compared to similar MR projects performed in collaboration with GPs, in which 77–93% of the recommendations were found to be implemented [26,31]. Hence, one can question why the implementation rate was so low considering that the GPs accepted the medication changes at the MR meeting. Thus, it could be interesting to clarify reasons for the lack of implementation.

In order to evaluate the potential of the use of symptom assessments as a future outcome of deprescribing interventions, we analyzed the change in symptoms between baseline and follow-up, i.e., before and after MRs, in three different ways: the mean change in symptom score; the number of patients with a deterioration or improvement of symptoms; and the identification of the total number of patients suffering from each symptom before and after the intervention. The changes in symptom scores were correlated with the changes in QoL.

Although not statistically significant, there was a clear trend towards an improvement of most symptom scores for the patients in the Deprescribing Subgroup between baseline and follow-up (Figure 2). This also applied to the overall Follow-Up Group, which, however, showed a weaker tendency for improvement than the Deprescribing Subgroup.

We deprescribed pain relievers for 12 patients, and 11 of these patients showed no deterioration in pain scores, indicating that they did not benefit from the pain relievers. This observation is in line with a recent Danish guideline, which does not recommend pain relievers (NSAID, paracetamol, opioids, and muscle relaxing drugs) for chronic use in non-cancer patients due to the lack of evidence of effects and the risk of increased adverse reactions for these patients [32,33]. Furthermore, seven of these twelve patients reported less pain after deprescribing pain relievers, which may indicate that the pain relievers might even have contributed to maintaining the pain, i.e., medication-induced pain [34,35,36].

The observed trends for symptom score assessments were supported by the QoL results of the Deprescribing Subgroup. Thus, in this group a tendency towards an improvement of QoL was found, which was not seen in the overall Follow-Up Group. For the Follow-Up Group, the EQ-5D index decreased after MR, but the VAS-score was improved (Table 2). Thus, the results for the change in QoL of the Follow-Up Group pointed in opposite directions, suggesting no overall change in QoL. Hence, the results of our study indicated a trend towards an overall improvement in both symptoms and QoL outcomes in the Deprescribing Subgroup (Figure 2, Figure 3 and Figure 4). However, apparently due to the lack of a critical mass in the study, the results did not show significant differences between baseline and follow-up values.

In summary, based on these results, symptom assessment may in the future be considered as a potential sensitive and specific method to assess an effect of deprescribing, i.e., either benefit or harm.

### Strengths and Limitations

This study secured a broad representation of nursing home residents and GPs, i.e., 3 different nursing homes and 18 GPs were included. In this study we had the advantage of direct access to the nursing home record. This made it possible to obtain an accurate picture of the patients’ current medication and the conditions regarding the individual medication administrations (both at baseline and follow-up), as well as limiting the loss-to-follow-up. Only the patients who died did not have a follow-up on the implementation of recommendations. The medication team consisted of two different professions to ensure high-quality recommendations, which was confirmed by the GPs’ high degree of acceptance of the suggested recommendations. For the symptom assessment and QoL, we ensured high quality and international applicability by using the validated, internationally recognized and ESAS-r and EQ-5D questionnaires, translated into Danish.

This study had some limitations worth noting, including the small number of patients, the low implementation rate and the failure to complete all questionnaires. A small number of patients and no control group is a consequence of being a descriptive study, and underline that the results of this study cannot be generalized but rather should be an inspiration for future work. The 3-month follow-up period was chosen to ensure that the patients’ state of health could be considered comparable in relation to their baseline score, to ensure a low loss-to-follow-up and at the same time to be long enough to enable us to assess the possible effect of deprescribing. However, a three-month follow-up period might have reduced the impact seen by deprescribing and multiple later follow-ups might be useful. In this study we saw a loss-to-follow-up of 7% due to death, but a further 7% of the initially included patients died before the intervention. A low implementation rate and the lack of completion of questionnaires might lead to selection bias, e.g., if the GPs only implemented recommendations in some intentionally selected patients or if only the patients with few symptoms were able to complete the questionnaires. In addition, the incomplete degree of implementation and completion of questionnaires suggested division into three post-hoc groups instead of prospective definition of groups. This is a further limitation of the study, which unfortunately reflects real world difficulties in conducting studies that include the present study population, i.e., frail elderly nursing home residents with high morbidity, and which involves interdisciplinary collaboration between physicians and nursing homes.

## 4. Materials and Methods

### 4.1. Design

This study was performed as a feasibility study based on an experimental design aiming at building initial understandings about symptom assessment as outcomes of medication reviews. The project was performed in a collaboration between the Department of Clinical Pharmacology at Copenhagen University Hospital Bispebjerg, Hvidovre Municipality, and the GPs of the included patients. Nursing homes in Hvidovre Municipality, Capital Region, Denmark, were offered MRs for elderly patients with polypharmacy. Patients were recruited in 2019, the MRs were conducted between March 2019 and February 2020 and the last follow-up was in May 2020. All patients were screened.

The MRs were prepared by an interdisciplinary medication team from the Department of Clinical Pharmacology at Copenhagen University Hospital Bispebjerg, consisting of a pharmacist and a medical doctor. The medication team visited each patient’s GP and together they conducted the MR with a duration per patient of 10 min. During the MR, the medication team recommended changes to the patient’s medication, e.g., deprescribing. Symptoms and QoL were measured before the MR and at follow-up (3 months after MR). A follow-up was performed by lookup in the individual care giver records after 3 months to allow time for the GPs to implement recommendations and for recommendations to impact symptoms and QoL, while preventing an excessive decline in the health state of these frail patients. The implementation of recommendations that were agreed upon by the GPs was evaluated at follow-up. The study design adheres to the Pharmacist Patient Care Intervention Reporting checklist:PaCIR [37] and the CONSORT extension for randomized pilot and feasibility trials used for non-randomized trials as proposed by Lancaster and Thabane [38].

### 4.2. Settings and Patients

The patients were recruited from the three public nursing homes in Hvidovre Municipality. Written informed consent was requested from the patients or their legal guardians to participate. Because we wanted to examine MRs in elderly polypharmacy patients, we included patients aged 65 years and older, who were prescribed five or more regular medications, including calcium tablets and strong vitamins (strong vitamins as defined by the Danish Medicines Agency [39]).

### 4.3. Medication Review

Based on the caregiver record, the medication list and input from the caregiving staff, the medication team prepared the MR for each patient. During the MR preparation, the medication team assessed the drug substance, dose, dosage form, dosage time and potential interactions for each medication in relation to the stated medications, indications and diagnoses. To assess the appropriateness of each drug, we used several decision tools in addition to national and regional guidelines, e.g., List of first choice medications, Capital Region, Denmark [40], Danish Deprescribing List [41], List of anticholinergic medicines [42] and National Database on Drug Interactions [43]. Each drug was assessed for the individual patient, and the same drug could be considered appropriate for some patients and inappropriate for others.

The medication team met with the patient’s GP and discussed the medication. On the basis of the medication team’s MR preparation and the newly acquired information from the GP, the medication team on site recommended changes to the patient’s medication with a specific focus on deprescribing. The GP could either accept or reject each recommendation. However, the final decision and implementation was a shared decision between the patient and their GP, which took place after the meeting with the GPs.

The recommendations were categorized into six types: Discontinuation; reduction of dose; increase of dose; change to another drug; change of dosage time; and reduce pill burden. Discontinuation included both abrupt discontinuation and tempering, depending on national/regional recommendations. In this article we focus especially on deprescribing, which covers both discontinuation and reduction of dose.

### 4.4. Outcomes

#### 4.4.1. Symptoms and QoL

Symptoms were measured using the Edmonton Symptom Assessment System (revised version) (ESAS-r) Scale [20]. The ESAS-r is a patient-reported instrument for patients near the end of life and is mostly used for monitoring symptoms in palliative care and hospices, but has also been used for monitoring symptoms in long-term care facilities [44,45,46,47] and to guide changes in the treatment of frail elderly patients [48]. The ESAS-r is based on a short numerical symptom scoring framework to enable quantitative measurements of symptoms with minimal patient burden [45,49]. The ESAS-r measures the intensity of each of eight symptoms (pain, tiredness, loss of appetite, nausea, depression, drowsiness, anxiety, and shortness of breath) using a numerical scale from 0 (no symptoms) to 10 (worst intensity of symptoms). ESAS-r also measures wellbeing using a numerical scale from 0 (best wellbeing) to 10 (worst wellbeing). ESAS-r measures multiple different symptoms that are typical for patients in the end of life, and symptoms which might be improved or deteriorated by medication. Deprescribing via an MR is an intervention without a specific medication or symptom target, and it may impact several symptoms. Therefore, ESAS-r may be suitable to evaluate the impact of deprescribing different medications. For each of the symptoms, the numerical value of 1 was used as the minimally clinically relevant difference for both improvement and deterioration [50]. In addition, we considered using the Neuropsychiatric Inventory (NPI) to assess the neuropsychiatric symptoms of patients with dementia [51]. However, this measurement would only assess behavioral disturbances of dementia and not physical symptoms. In addition, it would increase the patient burden. Thus, we decided not to use it in this study.

QoL was measured using EQ-5D. EQ-5D is a validated [21] extensively used, generic, non-disease-specific health-related QoL instrument, which is often used in the elderly population [9,13,52,53,54]. We used the five-level version of the EQ-5D instrument (EQ-5D-5L) which consisted of two sections. The first section—the EQ-5D index—consisted of five questions about health, which each had five ratings: mobility; self-care; usual activities, pain/discomfort; and anxiety/depression. The answers were converted (using the crosswalk index value calculator [55]) to a single score with values ranging from 1 (perfect health) to −0.59 (the worst imaginable health state), with the value of 0 indicating death. Furthermore, the EQ-5D has a visual analogue scale (VAS) which captures the responders’ self-ratings of their health from 100 (perfect health) to 0 (worst possible health). In this study, the minimum clinically important difference of 0.03 was used for the EQ-5D index and 8 for the VAS score [19,56].

We examined the following outcomes regarding symptoms and QoL: the mean change for each symptom between baseline and follow-up; the number of patients with a deterioration of each symptom; the number of patients with an improvement of each symptom; the number of patients with any representation (1–10) of each symptom; and the mean change in QoL. QoL was compared with the results of the symptom assessment to examine if the symptom assessment supported QoL measurements and/or contributed further relevant information.

#### 4.4.2. Medications and Recommendations

We examined the percentage of recommendations accepted by the GPs and the percentage of medications the GPs agreed to deprescribe to evaluate the relevance of the recommendations according to the GP and the perceived overmedication at baseline, respectively. In addition, the number and percentage of implemented deprescribing recommendations at follow-up were measured, to evaluate the MR’s effect on the medications.

### 4.5. Ethics

This project was approved by the Danish Data Protection Agency (I-Suite no 05564). According to Danish law, approval by the Danish Council on Ethics was not required and could not be obtained for this study, as we only recommended changes to the medication. The GPs decided which changes to accept and implement as part of their normal care for the patients. Each included patient or their legal guardian provided written informed consent.

### 4.6. Analysis and Statistics

Data were analyzed using SAS Enterprise Guide software (version 7.15. Copyright © 2022 SAS Institute Inc.). The data were collected in REDCap (Research Electronic Data Capture), which is a secure database approved for that purpose by the Danish Data Protection Agency [57].

Not all patients had completed the symptom assessment and QoL questionaries both at baseline and follow-up. Thus, we formed an investigational group including those who did (the Follow-Up Group).

To elucidate the symptom assessment for patients who had medication deprescribed, we created a subgroup (the Deprescribing Subgroup) consisting of only the patients from the Follow-Up Group who had a deprescribing intervention implemented at the follow-up.

Due to the descriptive nature of the study, we only included the most basic statistics to describe the results. The characteristics of the patients, divided into the different investigational groups (Table 1), were summarized and standard deviations were included for symptoms and QoL. For the outcomes, we analyzed the change with a paired *t*-test. For outcomes presented in tables, we included 95% confidence intervals, as they are more descriptive than a *p*-value, and we did not intend to show a significant change. For other outcomes, any significant change was mentioned in the text.

## 5. Conclusions

In this study we used the ESAS-r for symptom assessment as an outcome of medication reviews, with a focus on deprescribing. To our knowledge, this is a new approach. We observed a non-significant tendency towards improvement in most symptoms after deprescribing medication. This correlated with the tendency observed in QoL for these patients. The ESAS-r can be analyzed in different ways. However, we did not observe any clinically relevant effect on symptoms in patients who received deprescribing. Based on our results, we can conclude that symptom assessment is feasible and has potential as a valid outcome measure of deprescribing when performing MRs of elderly patients in nursing homes. However, an effort must be made to optimize the completion of questionnaires, either by improving the inclusion of healthier patients (e.g., home-dwelling elderly patients), or by ensuring caregivers are committed to helping the patients. A special focus should be directed towards an increased degree of implementation of recommendations.

In conclusion, our results indicate that symptom assessment may be a valuable, clinical and patient-relevant outcome of deprescribing studies, that is considered suitable for study upscaling and for use in other settings, e.g., home-dwelling elderly patients. This should be further elucidated in a randomized clinical setting with sufficient statistical power.

## Figures and Tables

**Table 1:** Characteristics of the patients constituting the investigational groups—the Medication Review Group, Follow-Up Group and Deprescribing Subgroup—regarding age, gender, medication, and outcomes. The symptoms and quality of life (QoL) questionnaires were not completed by all patients belonging to the Medication Review Group, which is why this column is not completed (-).

Investigational groups	**Medication Review Group, i.e., Patients Who Received MRs (*n*= 126)**		
		**Follow-up Group**, i.e., patients who completed the symptoms and QoL questionnaires (*n* = 86)	
			**Deprescribing Subgroup**, i.e., patients from the Follow-Up Group who had a deprescribing recommendation implemented (*n* = 48)
Mean age/year (SD)	82.3 (7.8)	81.8 (7.0)	82.2 (7.1)
Male/%	32%	30%	25%
**Medications at baseline**			
No. of medications	1.575	1.084	670
Mean no. of medications per patient	13	12	14
No. of recommendations	491	315	221
Mean no. of recommendations per patient	3.9	3.7	4.6
**Symptoms at baseline**			
Pain (SD)	-	2.9 (2.7)	3.3 (3.0)
Tiredness (SD)	-	4.2 (2.8)	4.5 (2.8)
Drowsinesss (SD)	-	3.3 (2.8)	3.5 (2.8)
Nausea (SD)	-	0.5 (1.5)	0.5 (1.2)
Loss of appetite (SD)	-	1.6 (2.4)	1.5 (2.3)
Shortness of breath (SD)	-	1.4 (2.3)	1.2 (2.2)
Depression (SD)	-	2.1 (2.7)	2.2 (2.7)
Anxiety (SD)	-	1.7 (2.6)	1.9 (2.8)
Wellbeing (SD)	-	3.3 (2.2)	3.3 (2.2)
**Quality of life at baseline**			
EQ-5D index (SD)	-	0.5 (0.3)	0.5 (0.3)
VAS score (SD)	-	56.2 (21.4)	56.2 (19.9)

**Table 2:** The mean difference in quality of life (QoL), the EQ-5D index, and VAS score, respectively, from baseline (before the medication review (MR)) to follow-up (3 months after the MR). The mean difference is shown for patients in the Follow-Up Group (*n* = 86), who completed the QoL questionnaire at baseline and follow-up, and for the Deprescribing Subgroup (*n* = 48), respectively. For the EQ-5D index and VAS score, a higher score indicates a better QoL.

Patient Group	EQ-5D IndexMean Change Higher Better (CI 95%)	VAS Score Mean Change Higher Better (CI 95%)
Follow-Up Group (*n* = 86)	−0.002 (−0.036: 0.176)	1.5 (−3.8: 6.8)
Deprescribed Subgroup (*n* = 48)	0.019 (−0.034: 0.073)	6.4 (−0.7: 13.5)

## Data Availability

Data is contained within the article.
